# Thyroid Cancer—The Tumor Immune Microenvironment (TIME) over Time and Space

**DOI:** 10.3390/cancers17050794

**Published:** 2025-02-26

**Authors:** Juan Carlos Jaume

**Affiliations:** 1 Department of Medicine, Edward Hines Jr. VA Hospital Hines, Hines, IL 60141, USA; juan.jaume@va.gov or jjaume1@luc.edu; 2 Department of Medicine, Loyola University Chicago, Chicago, IL 60660, USA

**Keywords:** thyroid cancer, tumor immune microenvironment (TIME), tumor-associated lymphocytes (TALs), myeloid-derived suppressor cells (MDSCs), tumor-associated macrophages, doble negative T cells (DN T cells)

## Abstract

In thyroid cancer, the tumor immune microenvironment (TIME) plays a crucial role in cancer development, progression and response to treatment. Like many other cancers, thyroid cancer creates a complex network of interactions with immune cells directly (cell-to-cell) and via humoral mediators (i.e., cytokines). This dynamic microenvironment undergoes constant modification, which can lead to changes in the immunophenotype that might explain cancer progression, dedifferentiation and resistance to treatment. According to the cancer immunoediting hypothesis, cancerous tumors can shape their immune microenvironment to create an immunosuppressive milieu that allows them to evade classic immune surveillance. One mechanism by which this occurs is through the reprogramming of immune cells, often shifting their phenotypes from cytotoxic to regulatory. Recent research has shed light on cellular components and molecular interactions within the thyroid cancer TIME. Immune cells such as Tumor-Associated Lymphocytes (TALs), myeloid-derived suppressor cells (MDSCs), Tumor-Associated Macrophages (TAMs) and Double-Negative (DN) T cells seem to play key roles in shaping the immune response to thyroid cancer. Additionally, cytokines, chemokines and other signaling molecules contribute to the communication and regulation of immune cells within that microenvironment. By studying these interactions, researchers aim to uncover not just potential therapeutic targets but also biomarkers of thyroid cancer that could provide clues on severity and progression. Based on that knowledge, strategies such as the use of immune checkpoint inhibitors, antigen-specific targeted immunotherapies, and immunomodulatory agents are being explored to enhance the anti-tumor immune response and overcome cancer immunosuppressive mechanisms. In this review, we analyze the available literature and provide our own experience to unravel the complexity of the thyroid immune microenvironment. Continued research in this area holds promise for improving outcomes through the identification of immune markers of severity/progression of thyroid cancer and the development of innovative immunotherapeutic approaches.

## Simple Summary

Immune surveillance of cancer cells is thought to prevent cancer development. Most clinically evident cancers do show immune cell infiltrates surrounding the tumor (“reactive lymphocytosis”) in pathology specimens. More than 50 years ago, it was shown that highly immunogenic tumors can spontaneously prime protective immunity, thus preventing tumor growth, a phenomenon known as tumor immunity. Current immunotherapeutic approaches for cancer treatment unleash tumor immunity by allowing for the expansion of cytotoxic immune responses. Researchers have sought to characterize the thyroid cancer immune microenvironment and compare it with immune responses in thyroid autoimmunity through development and progression. We discuss here the many immune players and their interaction throughout cancer development in the quest to come up with new target-specific immunotherapies.

## 1. Introduction

In thyroid cancer, the tumor immune microenvironment (TIME) plays a crucial role in cancer development, progression, and response to treatment. Like many other cancers, thyroid cancer creates a complex network of interactions with immune cells directly (cell-to-cell) and via humoral mediators (i.e., cytokines). This dynamic microenvironment undergoes constant modification, which can lead to changes in the immunophenotype that might explain cancer progression, dedifferentiation and resistance to treatment.

The concept of cancer co-existing with impaired immune surveillance of the tumor microenvironment is well established [1]. More than 50 years ago, it was shown that highly immunogenic tumors can spontaneously prime protective immunity in their microenvironment, thus preventing further growth, a phenomenon known as *tumor immunity* [2]. The generation of T-cell memory against tumor antigens may be a key to generating durable long-lived tumor protection [3]. However, poorly immunogenic tumors do not spontaneously induce a microenvironment of tumor immunity, nor do they prime functional T-cell responses [4]. Such tumors represent a large proportion of cancers in humans, where anti-tumor T-cell responses are often detected but do not control progression and may even favor tumor growth [5,6].

According to the cancer immunoediting hypothesis, cancerous tumors can shape their immune microenvironment to create an immunosuppressive milieu that allows them to evade classic immune surveillance [7]. A mechanism by which this occurs is through the reprogramming of immune cells, often shifting their phenotypes from cytotoxic to regulatory.

Autoimmunity on the other hand, is an effective form of cytotoxic immunity [8]. While known to immunologists for decades, it has surfaced in recent years largely due to accumulating evidence that, more effective immunotherapy of cancer is associated with autoimmunity [9,10]. In cancer development, progressive accumulation of genetic abnormalities renders cells malignant. The immune system seems to be allowing or even promoting cancer progression for some tumors [11]. While immune regulation in cancer seems to uphold development and progression, immune dysregulation in autoimmunity leads to (albeit expected) tissue destruction and eradication of the target (cytotoxicity). We have recently shown that full-blown autoimmunity to the thyroid gland (hypothyroid Hashimoto Thyroiditis and hyperthyroid Graves Disease) is mostly not associated with thyroid cancer [12]. Conversely, clinically silent subtypes of chronic inflammation (euthyroid forms of thyroiditis), appear significantly associated with thyroid cancer and may influence therapeutic responses [13]. Characterization of lymphocytic infiltrates of hypothyroid Hashimoto’s and hyperthyroid Graves’ glands (predominantly dysregulatory) demonstrated critical differences compared with those microenvironmental infiltrates accompanying thyroid cancer (predominantly regulatory) [12].

It is widely accepted that cancer development follows failure of immune surveillance. Cancer immune evading strategies outsmart sophisticated gene-mutation targeted therapies (precision/personalized cancer therapies and other attention-grabbing misnomers). New gene mutations occur within cancer cell populations as those cells are exposed to a selective drug pressure, where the cells with mutations that confer resistance are more likely to survive and reproduce, effectively “selecting out” the resistant cells while eliminating the susceptible ones. Ironically, just harnessing the naturally-existing immunity with immunotherapy appears to be a highly promising and maybe the only strategy for addressing cancer development/progression and its resistance strategies.

Here, we review the recent literature, provide our own expertise on the TIME in a cancer that develops in an immunity-prone gland (the thyroid) and speculate on the reprograming of immune regulatory cells into more cytotoxic ones to potentially treat cancer.

## 2. Materials and Methods

We searched the PubMed search engine for original studies published in English that addressed thyroid cancer, tumor immune microenvironment (TIME). The search terms were as follows: thyroid AND cancer AND microenvironment AND (immune cells OR autoimmune cells OR Hashimoto Thyroiditis OR Graves Disease). Relevant articles from our group, as well as those derived from the references of the query, were also analyzed. Studies were excluded if they did not specifically evaluate thyroid cancer microenvironment immune components. Abstracts were not included owing to our inability to completely assess validity and methodologies of the full studies. There were no date restrictions, and the search included 547 articles published through October 2024.

## 3. Results

Aside from our own group’s research, a total of 547 studies were captured in the initial query. Of those, 43 articles met the inclusion criteria stated as “addressing thyroid cancer, tumor immune microenvironment (TIME).” These 43 studies comprised articles that described cellular and molecular components of the TIME, in some cases as they related to thyroid autoimmunity (i.e., Hashimoto Thyroiditis). Some studies looked at the relevance of immune checkpoints’ presence in prognosis and treatment with inhibitors. Some described the differences between TIME of papillary versus anaplastic thyroid cancer. In the analyzed recent literature, when focusing on cellular components, more emphasis seems to be placed on macrophages (Tumor-Associated Macrophages, TAMs) than on regulatory T cells (Tregs). Of note, single-cell RNA analysis seems to provide a more comprehensive dissection of TIME, although cause and effect data is still missing.

### 3.1. Single-Cell Profiling

Single-cell RNA sequencing (scRNA-seq) is a technique that analyzes the RNA of individual cells. It is a powerful tool for studying the transcriptomes of cells, which can help identify rare cell populations and understand how cells interact. TIME cell profiling of human Papillary Thyroid Cancer (PTC) using scRNA-seq allows for analysis of cell lineages, transcriptional states, developmental trajectories, and cell-cell crosstalk in PTCs, thereby it has the potential of shedding light on the tumor ecosystems underlying PTC initiation and progression [14]. Even for evaluation of undetermined phenotypes, scRNA-seq could become a more definitive tool for diagnosis of ultrasound guided fine needle aspiration (USGFNA) biopsies [15]. When it comes to support cells like macrophages, scRNA-seq has been able to delineate mechanisms of M1 inhibition in PTC. Specifically, one study [16] identified the potential inhibition of macrophages via MIF signaling that allows for lymph node metastasis (see Section 3.4 for more on macrophages below). Immunosuppression has also been neatly described for anaplastic thyroid cancer using scRNA-seq [17]. Importantly, the differences between autoimmunity in Hashimoto Thyroiditis and PTC [18] and the possible links with thyroid cancer are being defined as well by these single-cell analyses [19]. Moreover, an interesting study from Wang et al. even established heterogeneity in bilateral PTC using scRNA-seq, solving the controversial hypothesis of intra-thyroidal metastasis [20].

In all these studies, cell sorting by specific phenotype (i.e., lymphocyte vs. dendritic cell vs. macrophage vs. thyroid cancer cell) prior to scRNA sequencing is essential for reliable analysis. Immediate processing without tissue culture manipulations is also crucial for functional interpretation. Moreover, scRNA-seq seems only reliable if hypothesis driven based on solid observation. Most importantly, longitudinal analysis as opposed to cross-sectional, is still needed for assessment of cancer progression.

### 3.2. Immune Checkpoints Analysis

Lymphocytes’ activation primarily relies on the specific recognition of antigens by antigen receptors, with the strength, duration, and nature of the activation signal often regulated by cell-surface receptor accessory molecules [8]. These immune checkpoints act as regulatory components, controlling timing and intensity of immune responses, and preventing immune hyperactivity in PTC [21]. These regulators inhibit immune responses, rendering the TIME incapable of mounting an efficient immune response against cancer, thus facilitating immune evasion and cancer progression. Common immune checkpoints in PTC include programmed cell death protein 1 (PD-1)/programmed cell death ligand 1 (PD-L1), and cytotoxic T lymphocyte antigen 4 (CTLA-4)/CD28 to a minor degree at least in PTC [22]. However, PD-1, PD-L1 or CTLA-4 in human clinical thyroid tumors are not prognostic for thyroid cancer outcomes. Many conclusions in literature are based on mouse models and might not be applicable to human cancer patients at this time.

#### Programmed Cell Death Protein 1/Programmed Cell Death Ligand 1

The PD-1/PD-L1 pathway has emerged as a vital cytotoxic T-cell (CTL) suppressive regulator in cancer. The overexpression of PD-L1 by cancer cells suggests that PD-L1 undermines immune surveillance in the TIME. Due to the cell-specific distribution of PD-L1 molecules present in tumor cells and PD-1 on CTLs, engagement of the two molecules leads to CTL apoptosis, with consequent cancer proliferation and distal invasion. When T cells recognized PD-L1-positive tumor cells, they engage the programmed T-cell death pathway. In addition, tumor cells can also produce cytokines including IL-10, allowing thyroid cancer cells to escape the clearance of CTL [21,22].

The PD-1/PD-L1 pathway represents one of the primary factor in cancer immune escape. Given their significance, PD-1/PD-L1 blocking agents (inhibitors) have shown considerable promise in cancer immunotherapy. In the specific case of Anaplastic Thyroid Cancer (ATC), its limited response to anti-PD-1 therapy seems due to low CTL abundance despite PD-L1 positivity in cancer cells. Additionally, some authors argue that PD-1 expression levels on CTL is low/absent in most ATC cases; a “target-missing” situation-unfavorable for an adequate therapeutic response [23]. However, there may be a way around it, as shown in an animal model of thyroid cancer, where pre-conditioning of the immune system by inducing chronic thyroiditis, favorably modulates the response of thyroid cancer to immune checkpoint inhibitors by increasing the number of PD-1+ CTLs in the TIME [24].

### 3.3. Myeloid-Derived Cells

Myeloid-derived cells, crucial players in anti-tumoral defense, are affected by tumor-derived factors and treatment. Some authors postulate that in the bone marrow, thyroid cancer patients tend to shift from myelopoiesis towards lymphopoiesis. Therefore, distinct transcriptional and functional changes in myeloid-derived cells arise before their infiltration of the tumor and are already present in the bone marrow, which suggests an active role of tumor humoral factors in forming the tumor immune microenvironment [25].

Others suggest that BRAFV600E (a common mutation in PTC) promotes thyroid cancer development by increasing myeloid-derived suppressor cells (MDSCs) penetrance [26]. This BRAFV600E-induced MDSC-mediated immune suppression involves re-activation of the developmental factor T-box transcription factor 3 (TBX3), which in turn, up-regulates C-X-C motif chemokine receptor 2 (CXCR2, a protein that regulates the migration and recruitment of lymphocytes) ligands, in a Toll Receptor 2 (TLR2)-Nuclear factor kappa-light-chain-enhancer of activated B cells (NF-κB) dependent manner, leading to MDSCs recruitment into the tumor microenvironment. This last hypothesis does not negate the myelo-to-lymphopoesis previously described but states that although not the dominant species, myeloid-derived cells may contribute to the TIME immunosuppressive capacity.

### 3.4. Macrophages

Although many groups have provided important contributions on tumor-associated macrophages (TAMs) as potential therapeutic targets in thyroid cancer [27,28,29,30,31,32], there are still many aspects to be discovered. We too explored the characteristics of macrophages in the TIME of thyroid cancer, particularly in the context of two thyroid conditions: Graves Disease (GD) and euthyroid Hashimoto Thyroiditis (EHT). We found that the immune cell composition and function in GD was somewhat different when compared with EHT. In Imam et al., 2019 [12], the focus was on understanding the dynamics between Natural Killer (NK) cells and macrophage (CD68+) polarization (M1 [CCR2+, CXCR1+] vs. M2 [ARGINASE1+, DECTIN1+]) in these two diseases’ backgrounds; and how these immune cells influence the progression of thyroid cancer. NK cells are innate lymphoid cells that mediate important effector functions in the control of viral infection and malignancy. Macrophages are also innate immune cells that have many functions, such as antigen presentation, phagocytosis of invading microorganisms, and clearance of cell debris. In GD, we documented higher proportions of NK cells and pro-inflammatory M1 macrophages co-existing in thyroid cancer TIME. NK cells in GD were highly activate, showing elevated levels of cytotoxic molecules (Granulysin, Granzyme B, Perforin) and INF-γ. M1 macrophages were abundant and played an active role in inducing tumoricidal activity as well [12]. However, in EHT (associated with thyroid cancer) lower NK cell and M1 macrophage presence, but higher M2 macrophage abundance was the norm. M2 macrophages, which generally support immunosuppressive responses, were more prevalent in EHT and contribute to a less effective TIME. Of note, a higher degree of macrophage plasticity was observed in EHT, meaning M2 macrophages were more likely to respond to immune stimulation and change their phenotype. M1 macrophages (classically activated and tumoricidal) were more prominent in GD and could reciprocally activate NK cells, enhancing their cytotoxicity. M2 macrophages (alternatively activated and pro-tumor) dominated in EHT, but were more plastic and could be reprogrammed to an M1-like phenotype when exposed to pro-inflammatory stimuli, such as lipopolysaccharide (LPS) or flagellin [12]. While the plasticity of macrophages in EHT allowed for reprograming, macrophages in GD were relatively more committed to a fixed M1 phenotype. Co-culturing activated NK cells with M2 macrophages led to upregulation of inflammatory cytokines (like IFN-γ) from NK cells, which in turn drove M2 macrophages towards an M1-like phenotype.

These findings suggest that immunomodulatory therapies targeting macrophage polarization could be beneficial in cancers that rely on M2 macrophages for tumor support, such as anaplastic thyroid cancer [32].

### 3.5. Double-Negative T Cells

In both mice and humans, about 1–5% of all peripheral T cells are of a double-negative (DN) phenotype [33]. These T cells do not express CD4 or CD8 cell surface molecules and show a characteristic cytokine profile. Zhang et al. [34] were the first to identify and characterize the immunoregulatory function of DN T cells. They demonstrated that murine DN T cells specifically eliminate activated anti-donor CD4+ and CD8+ T cells, and that adoptive transfer of DN T cells prolongs skin and heart allograft survival in transplant models. Human DN T cells can strongly suppress proliferation of CD4+ and CD8+ T cells too. Moreover, human DN T cells are able to downregulate cytokine production of highly activate human effector T cells [35]. As described in some of our studies [12,36,37], CD4+ and CD8+ T cells were significantly affected by the presence of DN T cells in thyroid cancer TIME. Importantly, patients with DN T-cell proportion >9.14% in the TIME, are at higher risk of carrying thyroid cancers [37]. Currently, there are no known transcription signatures which readily allow us to better define DN T cells. Therefore, DN T cells are likely overlooked in scRNA sequencing of cells in the thyroid cancer TIME. Okamura et al., 2024 [38], demonstrated significantly enriched DN T-cell populations in the tumor infiltrates of colorectal cancers which showed similar phenotype to central memory CD8+ T cells. Consequently, it is highly likely that current scRNA sequencing of clusters of T cells in CD8 compartments overlook the significant presence of DN T-cells in TIME. To correct this clustering bias, we scRNA sequenced, flow cytometry pre-sorted, DN T-cells from USGFNA of thyroid cancer TIME. DN T cells showed lower expression of T effector cell (Teff) activation marker genes, including GZmB, PFR1 and TNFRSF9 (CD137). They carried lower levels of exhaustion markers (LAG3, PD-1 and CXCL13) than CD8+ T-cells (similar to Zhu et al., observation, [39]), and higher expression level of naïve or memory marker genes (IL7R, SELL, TCF7 and FasL). Most distinctly, DN T cells expressed GZMK. Of note, GZMK is often associated with a complex role, potentially involved in immune regulation, and does not directly activate the apoptotic pathway like GZmB, the primary cytotoxic agent, directly triggering apoptosis by activating caspases within the target cell. The presence of GZMK in DN T cells confirms their regulatory nature.

## 4. Discussion

Recent research has shed light on cellular components and molecular interactions within the thyroid cancer TIME. Immune cells such as Tumor-Associated Lymphocytes (TALs), myeloid-derived suppressor cells (MDSCs), Tumor-Associated Macrophages (TAMs) and Double-Negative (DN) T cells seem to play key roles in shaping the immune response to thyroid cancer. Additionally, cytokines, chemokines and other signaling molecules contribute to the communication and regulation of immune and cancer cells within the thyroid cancer immune microenvironment.

By studying these interactions, researchers aim to uncover not just potential therapeutic targets but also biomarkers of thyroid cancer that could provide clues for severity and progression. Based on that knowledge, strategies such as immune checkpoint inhibition, antigen-specific targeted therapies, and immunomodulatory agents are being explored to enhance the anti-tumor immune response and overcome cancer immunosuppressive mechanisms.

Starting with supportive cells, others and we have underscored the complex interplay of macrophages and NK cells [12,40]. NK cells and macrophages, present in the immune microenvironment of thyroid cancer, interact in a different way than in the background of autoimmune thyroid diseases. As discussed above, in GD, the presence of activated NK cells leads to a higher M1/M2 ratio, which seems to offer protective immunity against cancer, while in EHT, the dominance of M2 macrophages, combined with lower NK cell activity, may contribute to a more permissive environment for tumor progression. However, the plasticity of M2 macrophages in EHT offers potential for therapeutic reprogramming; suggesting that strategies that enhance NK cell activity or promote M1 macrophage polarization could have a significant impact on cancer therapy, particularly in thyroid cancers that rely on M2 macrophage support like anaplastic variant [32].

The ability to manipulate macrophage polarization through immune stimulation may provide a new therapeutic avenue for thyroid and other cancers that are resistant to conventional therapies.

Another important key player now recognized in different cancers is the Double-Negative (DN) T cell. TCRαβ+CD56-CD4-CD8- DN T cells are known to be a minor sub-population of T cells, and their functions in TIME remain unclear.

The potential roles and characteristics of DN T cells for some cancers appear to be immunosuppressive. Several investigators suggest that some DN T cells may lack effector or cytotoxic functions, as observed in glioma and melanoma tissues TIME where they secreted immunosuppressive cytokine IL-10 [41]. Dominant TCRαβ pairs in DN T cells were identified that may competitively inhibit cytotoxic CD8+ T-cell responses by binding same tumor-specific antigens [42].

DN T cells show high GZMK expression, which may indicate pre-effector/memory T-cell characteristics. GZMK is known to play immunosuppressive roles, potentially also activating tumor-associated M2 macrophages. Significant correlations between GZMK expression and macrophage populations (M2) were observed in colorectal cancer RNA-seq data [43,44].

DN T cells in thyroid cancer are found in significantly higher proportion as opposed to thyroid autoimmunity [36]. We believe that these DN T cells likely originated from exhausted CD8+ T cells possibly through the downregulation of CD8 expression in activated and clonally expanded CD8+ T cells in the TIME.

**Figure 1:**
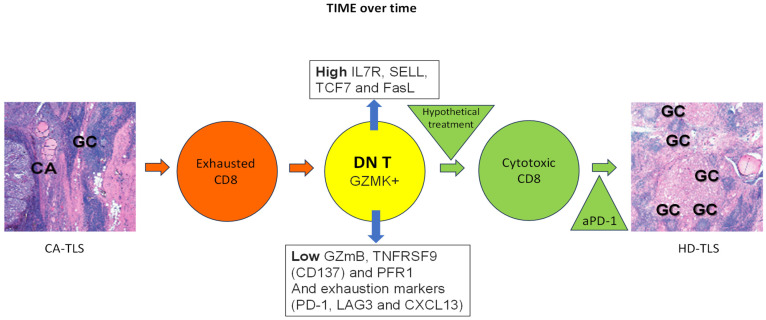
Tumor immune microenvironment (TIME) over time.

One possibility, following the immunoediting hypothesis of cancer (*elimination*, *equilibrium*, *escape*, 7), is that tumor immunity may result in a well-structured immune reaction in which T and B cells organize themselves in germinal centers (GCs) like the ones present in reactive lymph nodes or Hashimoto Thyroiditis (Tertiary Lymphoid Structures, TLS) which could eventually *eliminate* cancer. Indeed, incidentally discovered papillary thyroid microcarcinomas (as opposed to clinically detectable thyroid cancers) are more frequently found in patients with Hashimoto Thyroiditis [45]. Alternatively, cancer immunoediting may at times generate regulatory forces that immunosuppress effector cells (*equilibrium*, *escape*, 7), disrupting those well-organized TLS into loosely organized ones (cancer = CA-TLS; Figure 1).

Within cancer associated Tertiary Lymphoid Structure (CA-TLS) in the TIME of thyroid cancer (schematically represented with left side panel specimen, CA: cancer, GC: Germinal Center), exhausted CD8+ T cells (orange circle) may downregulate CD8 to become double-negative T cells (DN T, yellow circle) defined as positive for GZMK, low in GZmB, TNFRSF9 (CD137) and PFR1; and high in IL7R, SELL, TCF7 and FasL. Of note, the same exhaustion markers present in exhausted CD8+ T cells (PD-1, LAG3 and CXCL13) are also carried by DN T cells. Therefore, over time, the exhausted (because of continuous antigen presentation) CD8+ T cells might give origin to DN T cells (orange and yellow circles respectively). Treatment with immune stimulation is hypothesized may reprogram DN T cells into cytotoxic CD8+ T cells (green circle) and help shift the CA-TLS to a High CD8 Density Tertiary Lymphoid Structure, HD-TLS, in the TIME where for example, check point inhibitors (anti-PD-1) might help exacerbate cancer immune attack by CTLs (right side panel specimen HD-TLS, GC: Germinal Center).

There is consensus on the presence of CA-TLS in the TIME of thyroid cancer (left side panel specimen in Figure 1; CA: cancer; GC: Germinal Center). Others and we have shown the presence of exhausted CD8+ T cells co-existing with the dominant DN T cells defined as positive for GZMK, low in GZmB, TNFRSF9 (CD137) and PFR1 and high in IL7R, SELL, TCF7 and FasL (orange and yellow circles in Figure 1). Of note, the same exhaustion markers present in exhausted CD8+ T cells (PD-1, LAG3 and CXCL13) are also carried by DN T cells. Therefore, over time, the exhausted (because of continuous antigen presentation) CD8+ T cells might give origin to DN T cells. Treatment with immune stimulation is hypothesized to reprogram DN T cells into cytotoxic CD8+ T cells (green circle) and help shift the CA-TLS to a high CD8 density (HD)-TLS TIME where, for example, check point inhibitors (i.e., anti-PD-1) might help exacerbate a cancer immune attack by CTLs (right side panel specimen HD-TLS; GC: Germinal Center; all in Figure 1).

Considering the higher proportion of DN T cells suppress CD8+ T cells, through the Fas/FasL-mediated pathway in an antigen-specific manner, DN T cells might have direct immune-inhibitory roles in the thyroid cancer immune microenvironment as well.

Finally, DN T cells can be used for thyroid cancer screening and could provide prognostic value as immunogenomic markers [37]. As described, when DN T cells in the TIME are above a certain threshold, the chances of a tumor being malignant approaches 100%.

## 5. Conclusions

In this review, we have analyzed the available literature and shared our own experience to unravel the complexity of the thyroid cancer TIME. Researchers have identified the different immune players including MDSC, NK cells, macrophages, DN T cells and the interrelations that favor thyroid cancer development and progression. Evidence has now accumulated that cancer cells seem to negotiate their existence with the immune microenvironment, as predicted by the immunoediting hypothesis. New immune biomarkers for the evaluation of the severity/progression of thyroid cancer and new immune modulators of the TIME to drive tumor immunity are likely to be developed as a consequence of these advances.

## Data Availability

Our data can be found on PubMed by querying: thyroid AND cancer AND microenvironment AND (immune cells OR autoimmune OR Hashimoto Thyroiditis OR Graves Disease).
